# Single-cell and single-nuclei RNA sequencing as powerful tools to decipher cellular heterogeneity and dysregulation in neurodegenerative diseases

**DOI:** 10.3389/fcell.2022.884748

**Published:** 2022-10-24

**Authors:** Raquel Cuevas-Diaz Duran, Juan Carlos González-Orozco, Iván Velasco, Jia Qian Wu

**Affiliations:** ^1^ Tecnologico de Monterrey, Escuela de Medicina y Ciencias de la Salud, Monterrey, Mexico; ^2^ Instituto de Fisiología Celular—Neurociencias, Universidad Nacional Autónoma de México, Mexico City, Mexico; ^3^ Laboratorio de Reprogramación Celular, Instituto Nacional de Neurología y Neurocirugía “Manuel Velasco Suárez”, Mexico City, Mexico; ^4^ The Vivian L. Smith Department of Neurosurgery, McGovern Medical School, The University of Texas Health Science Center at Houston, Houston, TX, United States; ^5^ Center for Stem Cell and Regenerative Medicine, UT Brown Foundation Institute of Molecular Medicine, Houston, TX, United States; ^6^ MD Anderson Cancer Center UTHealth Graduate School of Biomedical Sciences, Houston, TX, United States

**Keywords:** single-cell sequencing, single-nuclei sequencing, Alzheimer’s disease, Parkinson’s disease, cellular heterogeneity, cellular vulnerability

## Abstract

Neurodegenerative diseases affect millions of people worldwide and there are currently no cures. Two types of common neurodegenerative diseases are Alzheimer’s (AD) and Parkinson’s disease (PD). Single-cell and single-nuclei RNA sequencing (scRNA-seq and snRNA-seq) have become powerful tools to elucidate the inherent complexity and dynamics of the central nervous system at cellular resolution. This technology has allowed the identification of cell types and states, providing new insights into cellular susceptibilities and molecular mechanisms underlying neurodegenerative conditions. Exciting research using high throughput scRNA-seq and snRNA-seq technologies to study AD and PD is emerging. Herein we review the recent progress in understanding these neurodegenerative diseases using these state-of-the-art technologies. We discuss the fundamental principles and implications of single-cell sequencing of the human brain. Moreover, we review some examples of the computational and analytical tools required to interpret the extensive amount of data generated from these assays. We conclude by highlighting challenges and limitations in the application of these technologies in the study of AD and PD.

## Introduction

Neurodegenerative diseases are characterized by a chronic and progressive structural and functional degeneration of cells in the central or peripheral nervous system, leading to massive neuronal loss. Unfortunately, there are currently no effective treatments for neurodegenerative diseases nor biomarkers for their early detection (Heemels, 2016). Data from the Global Burden of Diseases (GBD, 2020) demonstrates that Alzheimer’s and Parkinson’s diseases are the top neurodegenerative disorders contributing to the highest disability-adjusted life years (DALY) among the groups aged 75 or older (GBD 2019 Diseases and Injuries Collaborators, 2020). These common neurodegenerative diseases are predominantly idiopathic and have overlapping clinical and pathological features. Genome-wide association studies (GWAS) have identified genetic variants related to the risk of developing Alzheimer’s disease (AD) and Parkinson’s disease (PD). However, our understanding of the specific cell types dysregulated in these diseases remains elusive.

High throughput single-cell and single-nuclei mRNA sequencing (scRNA-seq/snRNA-seq) technologies have pioneered a new era of exploration into the diversity of brain cell types at the molecular level. Previously, the study of brain cells was limited to bulk assays in which the amount of RNA is averaged among all cells masking cell-type-specific gene expression (Colantuoni et al., 2011; Hawrylycz et al., 2012; Miller et al., 2014; Zhang et al., 2016). However, brain cells are heterogenous and functionally complex. ScRNA-seq/snRNA-seq technologies have provided an unprecedented opportunity to systematically investigate the expression of thousands of genes and cells, identifying cell subpopulations, and reconstructing temporal and spatial dynamics of gene expression. To this end, a comprehensive understanding of the brain cellular subtypes and their expression profiles in healthy physiological context and neurodegenerative conditions is required. Currently, scRNA-seq and snRNA-seq studies of mouse and human brains depict yields ranging from thousands of cells up to 1.3 million cells (Yao and van Velthoven, 2021). However, the scope of these studies can still be improved in the future given that the adult human brain has approximately 170 billion cells including a similar proportion of neuronal and non-neuronal cells (Azevedo et al., 2009). Exciting insights have been achieved with scRNA-seq and snRNA-seq in the field of human oligodendrocyte cell diversity (Marques et al., 2016), and functional states of human microglia (Masuda et al., 2019) among others. Furthermore, recent advances allow the correlation of molecular features with cellular processes such as proliferation leading to neurogenesis in the adult mouse hippocampus (Habib et al., 2016), as well as anatomical location and electrophysiological characteristics in the murine thalamus (Li et al., 2020).

Herein we will review the recent progress in understanding neurodegenerative diseases using single-cell sequencing. We will particularly focus on AD and PD. We will discuss the fundamental principles and implications of scRNA-seq and snRNA-seq of the human brain. Furthermore, we will briefly describe some examples of the computational and analytical tools used to interpret the extensive amount of data generated from these assays. Also, we will focus on pioneering human transcriptomic studies which have addressed the cellular heterogeneity of the brain regions mainly affected in AD and PD. Finally, we will highlight challenges and limitations in the application of these technologies in the study of AD and PD.

## Single-cell/nuclei transcriptomic experimentation

**FIGURE 1:**
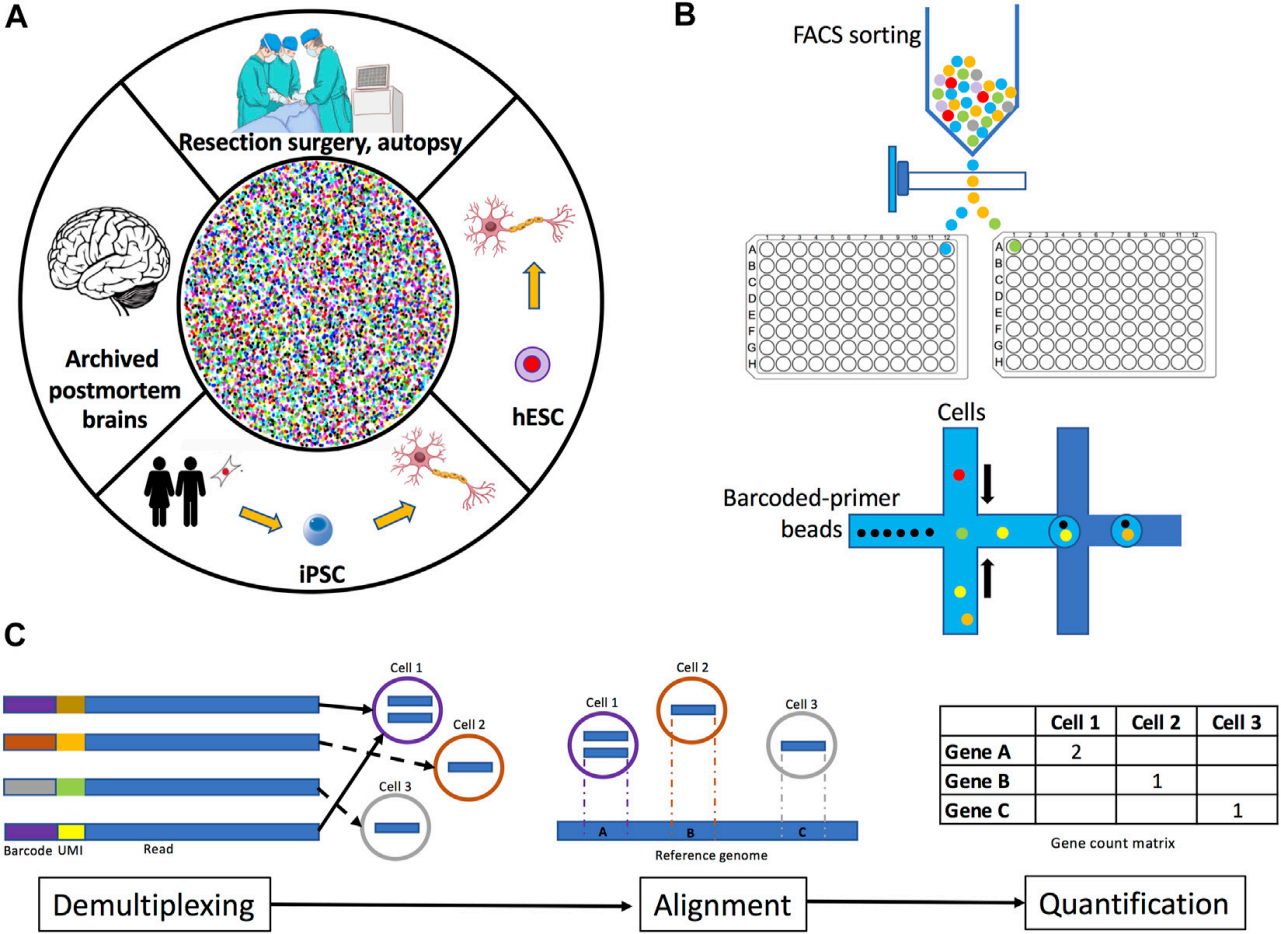
Overview of human brain single-cell and single-nuclei experimental methodology. **(A)** Tissue for isolating single-cells or single-nuclei may be obtained fresh from resection surgeries or autopsies, archived from frozen or formalin-fixed paraffin-embedded postmortem brains, or from neuronal cell cultures differentiated from iPSC or embryonic stem cells. **(B)** Plate-based and droplet-based single-cell and single-nuclei separation methods. **(C)** Data preprocessing methods include demultiplexing, read alignment, and quantification. Demultiplexing consists of the identification and removal of barcodes from sequencing reads. Read alignment is the process of mapping reads to a reference genome and/or transcriptome. Quantification involves the determination of the number of read counts per gene for each cell identified.

The first step in performing scRNA-seq/snRNA-seq is to identify the brain region of interest. Human brain cells or cell nuclei may be isolated from several sources: fresh tissue, archived organs, or from cells differentiated *in vitro* (Figure 1A). Samples from fresh tissue are collected from resection surgeries, biopsies, or autopsies (Gradišnik et al., 2021), while samples from archived organs are obtained from postmortem frozen or formalin-fixed paraffin-embedded brain sections (Wang et al., 2013). If frozen postmortem brains are selected, the best option is to collect the transcripts to be sequenced from the nucleus (snRNA-seq) because freezing ruptures the plasma membrane, thus complicating the isolation of intact cells and therefore increasing the probability of capturing degraded transcripts from the cytoplasm (Krishnaswami et al., 2016; Slyper et al., 2020; Maitra et al., 2021). Similarly, it is highly recommended to perform snRNA-seq instead of scRNA-seq on samples obtained from formalin-fixed tissues since the fixation process often damages the integrity of various cellular structures, leading to the detection of heavily degraded cytoplasmic RNA (Esteve-Codina et al., 2017). Furthermore, very recent protocols have optimized the extraction of cell nuclei for sequencing from formalin-fixed tissues, including brain (Chung et al., 2022; Vallejo et al., 2022). An alternative sample source is *in vitro* differentiation of either embryonic stem cells (ESCs) or reprogrammed induced pluripotent stem cells (iPSCs), which are a reliable source of biological material since the processes of artificial induction to the desired cell lineage highly recapitulates the molecular processes that occur in the organism during the development and maturation of cells (Marei et al., 2017). Moreover, some studies have already compared through scRNA-seq the transcriptomes of differentiated cells (e.g., dopaminergic neurons) from iPSCs and ESCs to the transcriptomes of cells obtained from human brain tissue to assess the fidelity of *in vitro*-derived cells, observing that these cells retain the characteristics from their *in vivo* counterparts (La Manno et al., 2016; Fernandes et al., 2020). Additionaly, some protocols optimized for scRNA-seq/snRNA-seq of *in vitro* differentiated cells recommend collecting the cells of interest by FACS sorting to increase the reliability of the procedure (Cuomo et al., 2020).

As mentioned above, the selection of the sequencing method may depend on the conservation status of the sample, but also on the type of cells to be analyzed. Single-cell isolation from brain represents a challenge because neurons are very sensitive to enzymatic and mechanical dissociation methods. Brain samples can be meticulously collected, for example using laser microdissection, and then carefully processed with an enzymatic or mechanical digestion method to obtain a single-cell suspension. However, as mentioned, there are at least two challenges when performing brain cell dissociation. First, since neuronal axons, dendrites, and synapses form an intricate and highly connected network in the brain, their dissociation may affect cellular integrity and even modify transcriptional profiles by upregulation of stress-induced artifacts (Denisenko et al., 2020). Second, neuronal transcripts residing in distal cellular compartments will most likely be lost upon dissociation (Cajigas et al., 2012; Tushev et al., 2018). These issues may lead to an underrepresentation of neuronal populations with respect to glial cells, which are more resistant to dissociation procedures as has been observed in single-cell suspensions obtained from human brain cortex (Darmanis et al., 2015). Therefore, careful consideration must be taken when selecting a cell dissociation method. Isolating single nuclei instead of single cells is a better option to analyze tissues that cannot be easily dissociated. Thus, an advantage of snRNA-seq is that it can capture transcripts from cells that are more susceptible to death in the process of dissociation for example, neurons from the brain cortex (Tasic et al., 2018). Moreover, nuclei isolation protocols are fast and do not require protease digestion or heating, reducing the probability of aberrant transcription (Lacar et al., 2016); also, nuclei protocols allow the profiling of large cells (<40 µm) that do not fit through microfluidics sequencing methods (Denisenko et al., 2020) and can be combined with gene regulatory studies, for example the assay for transposase-accessible chromatin sequencing (ATAC-seq). Despite all these advantages, an important concern for performing snRNA-seq is that nuclear transcripts represent only a percentage of the total cell’s mRNA, as demonstrated through a study of large and small pyramidal mouse neurons (Bakken et al., 2018). Notwithstanding, numerous studies (mostly in mice) have demonstrated that snRNA-seq is comparable to scRNA-seq; for example, although more transcripts were detected in individual whole cells than nuclei isolated from the primary visual cortex of mice, the independent analysis of the scRNA-seq and snRNA-seq datasets demonstrated that the same neuronal subtypes can be similarly discriminated with both sequencing methods (Bakken et al., 2018). Similar results were observed in another study where both sequencing methods were compared using samples isolated from mouse whole brain cortex (Ding et al., 2020), suggesting that snRNA-seq is useful and reliable for cellular diversity characterization of brain tissues. Hence, this method has been used extensively to profile and classify mouse brain cells (Yao *and Liu*, 2021). Regarding transcriptome coverage, researchers have demonstrated that snRNA-seq of mouse neural stem cells and hippocampal neurons detects 66% and 82% of the protein coding genes respectively, while the amount of gene expression variation was similar when measured between single nuclei and single cells (Grindberg et al., 2013). Furthermore, in human organs outside the nervous system such as kidney and pancreas, single-cell and single-nucleus methods yielded equivalent gene detection sensitivity with a significant percentage of transcripts detected in scRNA-seq and not detected in snRNA-seq corresponding to mitochondrial and stress-induced genes (Wu et al., 2019; Basile et al., 2021).

Once a single-cell/nucleus suspension is obtained, a method to physically separate them is used. Two common separation methods are plate-based cell sorting and droplet-based microfluidics (Figure 1B). In plate-based methods, cells or nuclei are isolated by fluorescence activated cell sorting (FACS) or nuclei sorting (FANS) into wells (Tasic et al., 2016) where lysis and library preparation are performed. With this method, samples can be stained to enable the exclusion of dead cells or enrichment of cells labeled with specific antibodies against cell surface or intracellular markers (Baran-Gale, Chandra and Kirschner, 2018). Droplet-based methods separate cells into nanoliter-size lipid droplets, where cells are lysed and library preparation takes place (Macosko et al., 2015). Library construction consists of capturing transcripts, labeling them with cell or nuclei-specific barcodes, reverse-transcribing them into cDNA, and amplifying them. Additionally, protocols may also label captured transcripts with unique molecular identifiers (UMIs) before amplification, this approach results in more accurate quantification of counts (Zheng et al., 2017). UMIs are random oligonucleotide barcodes of a fixed length and they are used to tag original transcripts and distinguish them from PCR duplicates (Islam et al., 2014). Droplet-based sequencing methods use lower reaction volumes and yield higher throughputs compared to plate-based methods (Forsberg et al., 2018). However, due to methodological differences, these technologies differ in the way they quantify transcripts. Droplet-based methods incorporate UMIs to either 5′ or 3′ ends, thus, they do not distinguish between gene isoforms. Contrastingly, plate-based methods capture the full-length transcripts including exons and splice junctions (Tasic et al., 2016; Gupta et al., 2018). Another decision to consider is the addition of spike-in RNA. Spike-ins are non-biological RNA molecules of known sequence which are added to each cell’s lysate in the same known concentration (Jiang et al., 2011). Spike-ins undergo all library preparation processes and thus they are very useful for normalization under the assumption that they are present in every cell in the same amount (Stegle, Teichmann and Marioni, 2015). However, the incorporation of spike-ins is not easy. For example, the concentration of the added spike-ins must be precisely calibrated to obtain optimal results, since small variations may lead to biased estimation. Spike-ins should be considered to contribute to 1%–5% of the total number of mRNA molecules in the sample (Robinson and Oshlack 2010; Grün and van Oudenaarden 2015). Furthermore, spike-ins are prone to degradation and may be captured less efficiently than endogenous transcripts in scRNA-seq/snRNA-seq experiments. Additionally, the inclusion of spike-ins is often complicated when using droplet-based separation methods (Haque et al., 2017; Svensson et al., 2017). Therefore, careful consideration should be taken when selecting the separation method.

## Key computational steps of single-cell/single-nuclei sequencing data analysis

Sequencing datasets, whether derived from single cells or nuclei are represented by matrixes in which rows correspond to features (e.g. thousands of genes or transcripts) and columns to barcodes (cells or nuclei). Data analysis may be challenging and requires the implementation of sophisticated computational methods some of which have different statistical assumptions (Kharchenko, 2021). Moreover, computational standards for single-cell/nucleus sequencing data analysis are still lacking, whereby researchers must be very careful when selecting the appropriate computational analysis methods. Excellent reviews focused on detailing the best practices for computational data analysis and interpretation have been published (Luecken and Theis, 2019; Andrews et al., 2021; Slovin et al., 2021). In this section, we will describe some of the more general steps required for analyzing scRNA-seq or snRNA-seq datasets (referred to as single-cell datasets because the same type of computational analysis is applied for both sequencing methods) and their downstream analysis.

### Data preprocessing

In this first data analysis step, sequenced reads are summarized into a count matrix. Generally, preprocessing pipelines include demultiplexing and barcode correction, alignment to reference genome or transcriptome, quantification, and quality control (Figure 1C). Some common preprocessing pipelines are CellRanger (Zheng et al., 2017) and Optimus (https://data.humancellatlas.org/pipelines/optimus-workflow) supporting 10x Chromium datasets, dropEst (Petukhov et al., 2018) and DropSeqTools (https://github.com/broadinstitute/Drop-seq) developed for droplet-based protocols, and zUMIs (Parekh et al., 2018) compatible with all UMI-based protocols. A benchmark of common preprocessing methods is described in (You et al., 2021). Demultiplexing consists on the identification and removal of barcodes from sequencing reads. In this process, reads are grouped together by barcode similarity. However, since synthetic techniques of barcode generation are prone to deletion errors, the next process is barcode correction. Corrupted barcodes undergo an error correction by comparing their sequences to a set of known barcodes, provided in the library preparation kit (Macosko et al., 2015). If the assay was performed including UMIs, demultiplexing will attach the UMI sequence to the read name. The number of different UMIs per cell is an important metric to distinguish empty droplets/wells and outliers (Islam et al., 2014). Finally, PCR duplicates are removed, and read counts are assigned to individual genes and cells.

**FIGURE 2:**
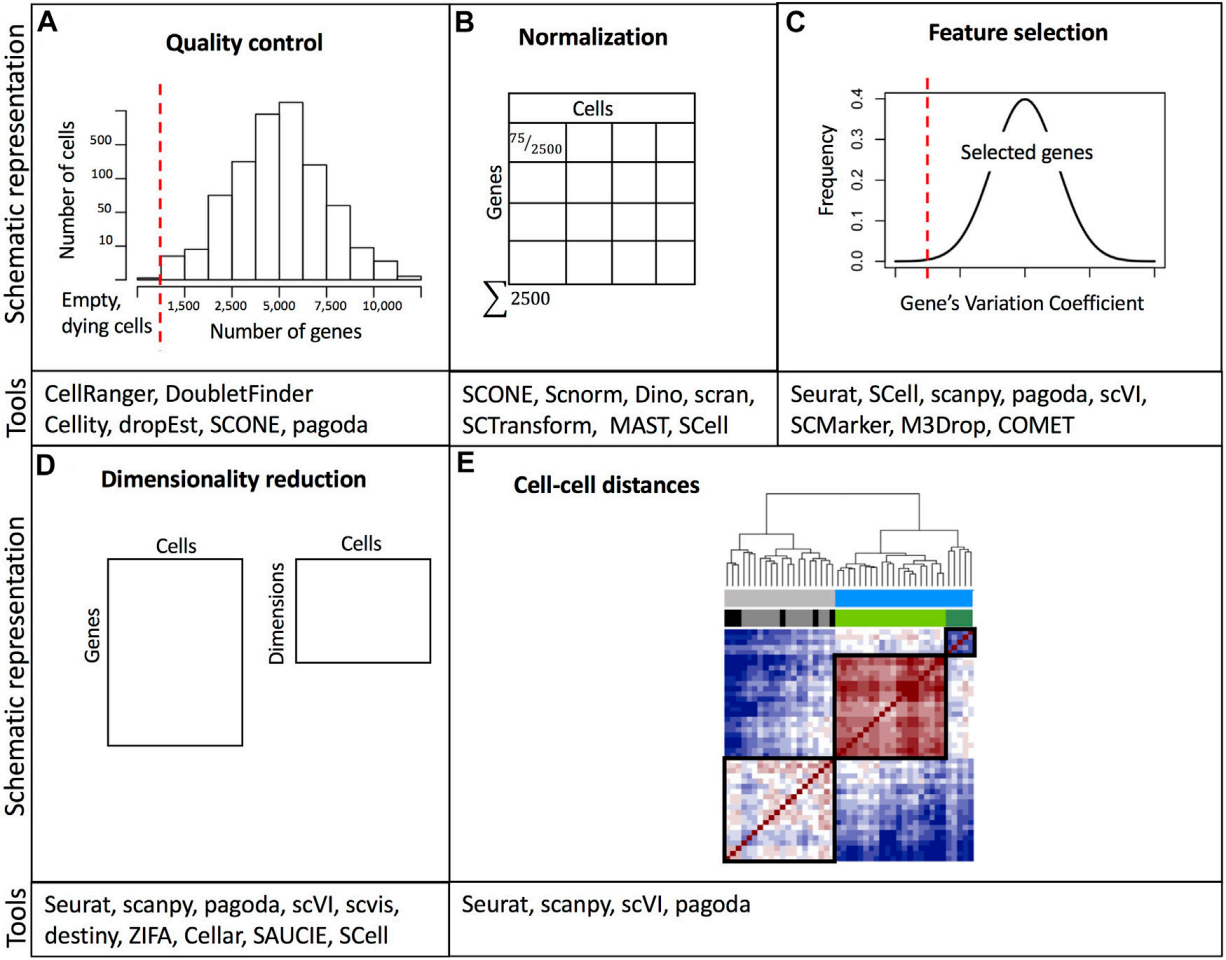
Overview of common data processing steps with examples of relevant software tools. **(A)** Example of threshold assignment for the minimum number of genes per cell as part of the quality control. Cells with a small number of detected genes are likely empty wells/droplets or non-viable cells. **(B)** Normalization of gene expression based on cell depth. **(C)** Filtering out genes with low variability is part of the feature selection. **(D)** Dimensionality reduction. **(E)** Calculation of a distance matrix based on cell-cell gene expression similarity metrics. A comprehensive list of software packages for single-cell data analysis is included in https://github.com/seandavi/awesome-single-cell. References to example tools are listed in Supplementary Table S2.

The last step in preprocessing is determining the quality of the captured cells/nuclei. Quality control considers three metrics: the number of counts per cell (barcode) also referred to as count depth, the number of genes per cell, and the fraction of counts per cell which mapped to mitochondrial genes (Ilicic et al., 2016; Griffiths, Scialdone and Marioni, 2018). Users assign thresholds to these metrics to filter out potentially non-viable cells or doublets (Figure 2A). It is important to note that threshold assignment for filtering cells should be selected cautiously since the behavior of these metrics may be of biological relevance. Cells with an elevated number of genes with a high number of counts are potential doublets. Doublets may occur when two or more cells/nuclei are captured in the same well or droplet. Computational methods for detecting doublets have been developed: Doublet Finder (McGinnis, Murrow and Gartner, 2019), Scrublet (Wolock, Lopez and Klein, 2019), DoubletDecon (DePasquale et al., 2019), and scds (Bais and Kostka, 2020). Another relevant aspect of quality control unique to droplet-based methods is the probability that some droplets will not be able to capture a viable cell or nucleus, but rather capture ambient RNA that was present in the cell suspension (Hong et al., 2022). These empty droplets must be filtered out for downstream analysis and some tools such as EmptyDrops or scCB2 have been developed to discriminate real cells from background barcodes (Lun et al., 2019; Ni et al., 2020). Finally, it is also important to consider that ambient RNA can also be present in droplets containing cells and can be detected in combination with endogenous cellular RNA, thus contaminating the single-cell dataset (Hong et al., 2022). For this purpose, computational tools including DecontX and SoupX can be used to estimate contamination levels and to separate endogenous RNA reads from ambient RNA reads (Yang et al., 2020; Young and Behjati, 2020).

### Normalization

Variations in cell lysis, reverse transcription efficiency, and stochastic molecular sampling during sequencing may generate differences in identical cells’ sequencing depths (number of detected genes per cell) (Hicks et al., 2018). Furthermore, single-cell datasets suffer from increased sparsity, which means a high proportion of zero read counts derived from both biological and technical reasons (Kharchenko, Silberstein and Scadden, 2014; Lun, Bach and Marioni, 2016). Thus, normalization methods aim at removing technical biases while preserving real biological variation. After normalization accurate relative gene expression abundances between cells are obtained and gene counts are comparable between cells (Figure 2B). There are two broad categories of normalization methods depending on whether spike-ins were added during library preparation. Data sets with spike-ins are generally normalized using the counts of these RNAs as a reference to scale cell counts assuming that the same number of these molecules was added to each cell. However, adding the same quantity of spike-ins to all cells is technically challenging, especially in droplet-based protocols. Thus, other normalization methods have been implemented. These other methods rely on the assumption that the majority of genes are not differentially expressed. In this sense, a commonly used global linear normalization method adopted from bulk RNA-seq analysis is count depth scaling. This method calculates counts per million (CPM) assuming that all sampled cells have an equal number of mRNA molecules. Nevertheless, this is insufficient and may bias comparisons when differentially expressed genes (DEGs) are present (Robinson and Oshlack, 2010). Instead, methods based on count ratios between cells are more robust to DEGs, for example, the median of ratios and the trimmed mean of M values (TMM) implemented in DESeq and EdgeR respectively (Robinson and Oshlack, 2010; Love, Huber and Anders, 2014).

Algorithms based on quantile normalization define scaling factors based on each cell’s count distribution (e.g. upper quartile) or by fitting to a reference distribution (e.g. full quantile). Still, these methods are biased due to the high proportion of zero counts affecting the count ratios and gene expression distribution (Vallejos et al., 2017). A method developed specifically for single-cell RNA-seq was proposed by Lun et al. to account for cellular heterogeneity and zero counts (Lun, Bach and Marioni, 2016). In this method, the authors used a strategy where expression values are summed across pools of cells for normalization, then cell-specific size factors are deconvolved from pool factors. This method is implemented in the scran R library (http://bioconductor.org/packages/scran) and it has proven to yield more accurate scaling factors than others because it reduces the incidence of zeros by counts summation across pooled cells (Lun, Bach and Marioni, 2016; Vieth et al., 2019). Other normalization methods not relying on global scaling factors have been proposed, for example, SCnorm (Bacher et al., 2017). In this approach, zero values are filtered out and then two stages of quantile regressions are used for normalization, one to group genes based on their dependence on sequencing depth and the other to estimate scale factors within each group (Bacher et al., 2017). However, it is important to mention that removing or reducing zeros from datasets may lead to biases (especially where a low-abundance gene is expressed in many cells), thereby ignoring potential information (Linderman et al., 2022). For this reason, some authors have proposed to evaluate relative gene expression by determining the percentage of cells with non-zero expression for a specific gene instead of using normalized gene counts where zeros were reduced or removed from the dataset (Booeshaghi and Pachter, 2021).

Non-linear normalization methods have also been developed to account for the multiple sources of variation in single-cell data (Cole et al., 2019). The majority of these algorithms attempt to model read counts according to a parametric distribution. Technical covariates such as read depth and counts per gene can be used to adjust the model. A commonly used non-linear normalization method was implemented in the R package SCTransform (Hafemeister and Satija, 2019). In this approach, authors fit cell counts to a negative binomial distribution and use sequencing depth as a covariate in their linear regression model. SCTransform outperforms global scaling methods and it has recently been included in Seurat, an R toolkit for single-cell genomics (https://satijalab.org/seurat/) (Satija et al., 2015). Another recently developed method based on negative binomial distribution is the R package Dino (Brown et al., 2021). In this method (specially designed for UMI-based protocols), normalization is performed by correcting the full expression distribution of each gene for library size dependent variation, rather than only correcting mean expression as most of the existing methods do, resulting in a robust approach for sample heterogeneity maintenance (Brown et al., 2021).

The distribution of gene counts is dramatically skewed with both an elevated fraction of zero counts and a reduced number of genes with high read counts. Thus, normalizing for gene counts (z-score) aims at improving gene comparisons within and between cells placing them in a similar scale. After normalization, a common practice is to transform data counts using a log(x+1) function. Although data counts do not follow a log-normal distribution (Vieth et al., 2017), this transformation is useful for downstream analysis, for example, differential expression. However, it has been demonstrated that log-transformation may introduce systematic errors leading to spurious differential expression effects (Lun, 2018). These artifacts are more dramatic when there are big differences between cells’ size factors and the read depth is low. Therefore, caution is suggested when interpreting clusters and trajectories derived from datasets with these conditions. Furthermore, it is also relevant to mention that a growing number of computational methods such as MAGIC, SAVER, or kNN-smoothing (van Dijk et al., 2018; Huang et al., 2018; Almanjahie et al., 2021) have been proposed to resolve the increased sparsity observed in single-cell datasets by imputing data (thereby named as imputation methods) to values that are missing or unobservable, thus improving the analysis of datasets with an elevated fraction of zero counts (Patruno et al., 2021). Some imputation methods directly address the sparsity of the single-cell datasets by using probabilistic models to distinguish biological from technical zeros and then adding values only to the technical ones. Meanwhile, other methods adjust all zero and non-zero values by smoothing or diffusing gene expression values in cells with similar expression profiles (Hou et al., 2020). Albeit imputation methods represent a potential alternative for single-cell analysis, they have the challenge of imputing data accurately while preserving true biological zeros. Also, although to date there are no quantitative benchmarks to evaluate such methods, recent studies has shown that some imputation methods can outperform non-imputation methods in recovering gene expression, however, imputation methods did not improve performance in downstream analysis, particularly in clustering and trajectory analysis, therefore these methods should be used with caution (Hou et al., 2020; Patruno et al., 2021; Linderman et al., 2022).

Typically, toolkits implement more than one normalization method. For example, Seurat’s LogNormalize function, uses a global-scaling normalization method by dividing counts for each cell by the total counts for that cell, multiplying by a scale factor (10,000 by default), and transforming data with a natural-log function (Satija et al., 2015). An overview of common normalization methods, their characteristics and limitations are depicted in Supplementary Table S1. The selection of the normalization method to use should be considered carefully since it highly impacts downstream analysis.

### Feature selection, dimensionality reduction, and visualization

Single-cell datasets have a high number of features (genes or transcripts). Many of these features or genes contain zero counts (“dropouts”) for the majority of cells or their count values are not informative towards explaining the biological variability. Dropout events do not signify a lack of expression, instead, they potentially represent a failure in the detection of a transcript (Qiu, 2020). Therefore, in the feature selection step, the researcher must decide which genes to keep (Figure 2C). Generally, the top most variable genes (1,000–5,000) are the ones that may be useful in explaining cell-to-cell variability (Luecken and Theis, 2019; Yip, Sham and Wang, 2019). After selecting the most variable features, the dimensionality of expression matrices (number of rows or genes) can further be reduced from thousands to less than 100 using linear or non-linear feature projections (Figure 2D). Several dimensionality reduction techniques are available, however, a popular one is principal component analysis (PCA). PCA performs linear combinations of genes that maximize the residual variance of such combinations. The top principal components (PC) explain the majority of the data variation. Each PC, also referred to as “metagene” is a linear combination of several genes. PCA is useful for downstream analysis (e.g., clustering), however since single-cell data is inherently non-linear, PCA is not the best option for data visualization, even though it is very efficient in reducing dimensions. PCA depicts limitations derived from the fact that the computation of the PCs is not related to the underlying statistical structure of single-cell data. Since single cells can have more zero read counts than others (due to technical factors), PCA may identify this difference as one of the top PCs (Kharchenko, 2021). Instead, manifold learning methods are preferred for dimensionality reduction and data visualization. A manifold is a topological or geometric space that locally resembles a Euclidean space. Moreover, the manifold hypothesis states that high-dimensional data lie in low-dimensional manifolds embedded in high-dimensional space (Fefferman, Mitter and Narayanan, 2016). Two commonly used manifold-learning methods for dimensionality reduction and visualization include t-distributed stochastic neighbor embedding (t-SNE) (Bonin-Font, Ortiz and Oliver, 2008) and Uniform Approximation and Projection Method (UMAP) (McInnes et al., 2018). In t-SNE, high-dimensional data are mapped into two dimensions allowing neighboring similar cells to remain close and distant cells to remain far in the low-dimensional space. t-SNE requires setting the parameter denominated “perplexity” to control the width of the Gaussian function used to determine the similarity between cells. Differing perplexity parameters may yield different number of clusters (Wattenberg, Viégas and Johnson, 2016). A caveat of t-SNE is that it fails to capture the global geometry of the data, fragmenting natural progressions or trajectories (Kobak and Berens, 2019). This is especially problematic when single-cell datasets were obtained from cell classes with a meaningful hierarchy, e.g. progenitor and differentiated cell subpopulations, and induction time points. Another commonly used alternative is the UMAP. UMAPs create a fuzzy graph from the data matrix to reflect its topology and then build a low-dimensional graph using the weight of the edges. UMAP has been demonstrated to provide the fastest run times as well as the ability to process large numbers of cells (Becht et al., 2018), and it is recommended as a best practice for exploratory data visualization since some studies have shown that it better preserves the global structure of the data (Becht et al., 2018; Luecken and Theis, 2019), albeit other studies indicate that UMAP has the same performance as t-SNE in preserving the global structure, and is only superior to this latter method in the initial implementations for data embedding (Kobak and Linderman, 2021). Finally, it is important to emphasize that both t-SNE and UMAP are only used for dimension reduction and data visualization and not for downstream analysis such as clustering.

### Clustering

**FIGURE 3:**
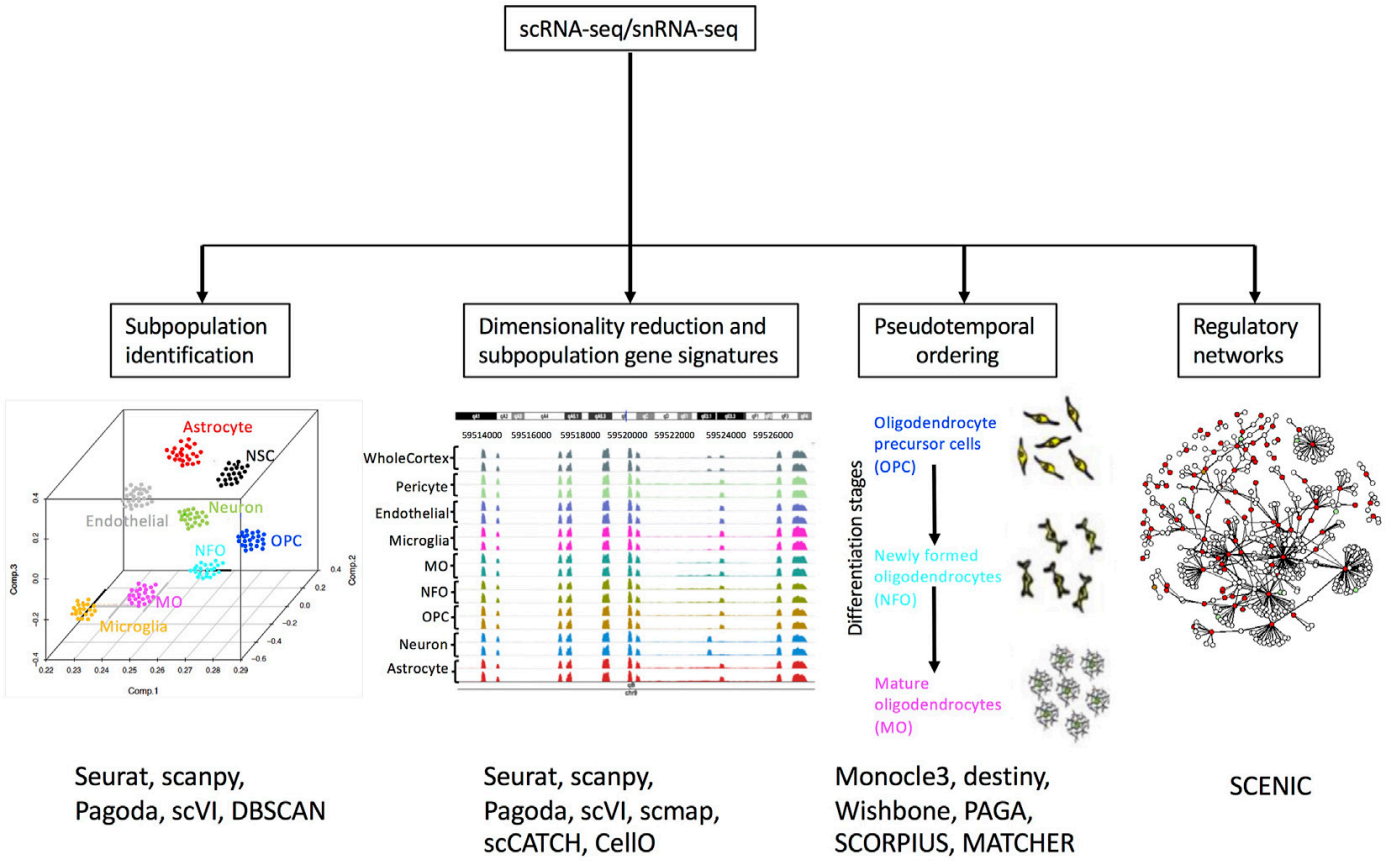
Overview of scRNA-seq and snRNA-seq downstream analysis. Examples of relevant software tools used for each application are included. References to example tools are listed in Supplementary Table S2. Adapted from (Cuevas-Diaz Duran, Wei and Wu, 2017) ^©^ Cuevas-Diaz Duran, Wei and Wu, 2017.

One of the most popular applications of single-cell transcriptomics is clustering or grouping cells based on transcriptional similarity (Figure 3). Clustering allows researchers to determine the number of subpopulations with distinct molecular signatures in a sample. Furthermore, based on expression profiles, researchers can infer cluster identities and arrange them in a hierarchy (Cuevas-Diaz Duran et al., 2017; Zeng and Sanes, 2017). Grouping cells based on similarity is performed using two approaches: clustering algorithms and community detection methods. Clustering algorithms group cells based on a distance metric (Figure 2E), however, due to the high dimensionality of data, distances between cells can be very similar. This is known as the “curse of dimensionality” (Bellman, 1961) and it may be overcome by feature selection and dimensionality reduction. Since t-SNE and UMAP methods highly compress multidimensional data in two dimensions, they are not recommended for clustering analysis. Moreover, it has been suggested that t-SNE and UMAP may introduce significant distortion to the data (Chari et al., 2021), although efforts have been made to combine PCA with t-SNE to make it more suitable for clustering (Kobak and Berens, 2019). Therefore, PCA or some of its variants like weighted PCA (WPCA) or SpatialPCA are highly used for dimensionality reduction prior to clustering (Tsuyuzaki et al., 2020; Liu et al., 2022). Clustering algorithms, based on unsupervised machine learning methods use a distance calculation, for example, Euclidean, cosine similarity, or Pearson’s and Spearman’s correlation, to determine intracluster distances. Cells are assigned to clusters where similarity distances are minimized. A common clustering method used is *k*-means, which iteratively assigns cells into *k* clusters where the distance from a cell to a cluster centroid is the lowest. Numerous applications of *k*-means clustering have been developed with different distance metrics (Grün et al., 2015; Kiselev et al., 2017; Hicks et al., 2021). *K*-means clustering requires the number of clusters as input, thus users may need to run several scenarios and determine the best choice. A shortcoming of *k*-means clustering methods is that they tend to identify round clusters of equal size, missing out on rare cell subpopulations (Kiselev, Andrews and Hemberg, 2019). To overcome the drawback of k-means clustering, community detection algorithms have been implemented successfully.

Community detection algorithms rely on representing single-cell data as graphs with nodes and edges. These methods construct *K*-nearest neighbor graphs (KNN) from a low-dimensional space. Cells are represented as nodes and they are connected by edges to their *K* most similar nodes using a distance metric, typically Euclidean. Densely connected nodes are identified as clusters through community detection methods, which are faster than other clustering methods and can scale up to millions of cells (Rosvall and Bergstrom, 2008). Community detection-based methods outperform *k*-means clustering methods in large-scale datasets, and one of the most commonly used is the Louvain community detection algorithm (Blondel et al., 2008). This method identifies communities under the premise that cells in each group will have more links between them than what is expected from the total number of links. In this way, the algorithm optimizes a modularity function (number of links) by iteratively assigning nodes to different communities (Blondel et al., 2008). Louvain community detection is implemented in SCANPY (Wolf, Angerer and Theis, 2018) and Seurat toolkit (Satija et al., 2015). Although the Louvain logarithm is very popular, some authors have identified flaws, resulting in badly connected communities, so the use of the Leiden algorithm has been proposed as an alternative (Traag et al., 2019). In contrast to Louvain, the Leiden algorithm can split clusters instead of only merging them; furthermore, by relying on a fast local move approach, the Leiden algorithm runs faster than the Louvain algorithm (Anuar et al., 2021) and it is also included in Seurat toolkit (Satija et al., 2015).

Density-based clustering methods identify cell clusters by recognizing contiguous regions of high-density of cells. These methods can identify cell clusters with arbitrary shapes and sizes. The most popular density-based clustering method is Density Based Spatial Clustering of Applications with Noise (DBSCAN) (Ester et al., 1996). These methods do not need an *a priori* number of clusters, however, density parameters must be provided. A caveat of these methods is that they assume that all clusters have similar densities. Nonetheless, DBSCAN was included in early versions of Seurat (Satija et al., 2015) and GiniClust (Jiang et al., 2016) for the detection of rare cell types.

Alternative approaches based on matrix decomposition have also been proposed, for example, Nonnegative Matrix Factorization (NMF). NMF has conventionally been used to decompose high-dimensional transcriptional data (genes) into interpretable features (meta-genes). Nevertheless, NMF has been successfully implemented to find clusters of cells from a samples-by-genes matrix (Shao and Höfer, 2017; Duren et al., 2018; Kotliar et al., 2019; Wu et al., 2020).

### Cluster characterization

Cluster annotation consists of finding a set of genes denoted as the “gene signature” for each cluster (Figure 3). It is important to note that the transcriptional differences found do not always correspond to cell types since other variables such as cell-state and cell-cycle can have a greater influence on transcription (Buettner et al., 2015). Furthermore, differences in the cellular state may cause cells of the same type to be assigned to different clusters. Thus, interpreting cell clusters is not trivial and researchers must rely on external reference databases, for example, the Human Cell Atlas (Regev et al., 2017) or, on the expression of marker genes derived from literature. DEGs may be obtained by comparing the gene expression of cells belonging to a cluster to all other cells. Due to the nature of unsupervised clustering methods, clusters will always depict DEGs (Luecken and Theis, 2019). Upregulated genes are ranked using statistical tests such as t-tests or Wilcoxon rank-sum test and the top-ranking genes are referred to as “marker genes” (genes that have a high expression only in one cluster). Then, these marker genes are manually compared to available information in the literature or in reference databases to annotate cell type labels for each detected cluster (Zhang et al., 2019). Nevertheless, the manual characterization of clusters is prone to error since marker genes are often expressed in multiple clusters and correspond to multiple cell types. In addition, negative marker genes should also be considered for the annotation of cell types (Ianevski et al., 2022). In this context, other approaches for cluster characterization rely on an automatic annotation of cell types (Nguyen and Griss, 2022). These computational methods are based on different principles. For example, scmap is a method that assigns cell type labels to clusters by projecting cells to cell type landmarks, then inferring unknown cells by proximity to already known cell types in the embedded space (Kiselev et al., 2018). Another approach is to correlate marker genes in annotated clusters of cells with unannotated clusters, this is implemented in the methods scCATCH (Shao et al., 2020), CIPR (Ekiz et al., 2020), ScType (Ianevski et al., 2022) and clustifyr (Fu et al., 2020). Meanwhile, other methods such as CellO use machine learning for cell type classification of cell clusters by considering the rich hierarchical structure of known cell types (Bernstein et al., 2020). A caveat on the use of marker genes in automated methods is that performance heavily relies on gene lists provided as markers for specific cell types that in some cases were manually constructed, or on marker databases that are still suboptimal both in coverage and specificity (Ianevski et al., 2022). More extensive reviews about methods for cluster characterization and cell type annotation providing guidelines and recommendations for their use are available (Clarke et al., 2021); Pasquini et al., 2021; Nguyen and Griss, 2022).

Finally, it is relevant to mention that DEGs are also used for functional enrichment analysis to identify biologically relevant functions. Importantly, marker genes representing each detected cluster should be used for experimental validation, for example, RT-qPCR, cytometry, or *in situ* imaging.

### Trajectory inference

An application of single-cell transcriptomics is trajectory inference. These computational methods are based on the premise that single-cell data is a snapshot of a continuous biological process that drives the observed heterogeneity (Trapnell, 2015). Cells are assigned a pseudo time or numerical value that measures the distance of a cell in a dynamic biological process, for example, a differentiation process (Cannoodt, Saelens and Saeys, 2016). Transition stages can be defined when cells are ordered according to their pseudo time, this is known as pseudo temporal ordering (Figure 3). Trajectory inference methods work by implementing a dimensionality reduction and a trajectory modeling step (Cannoodt, Saelens and Saeys, 2016). Trajectory modeling uses a graph representation of the data and finds a path that connects nodes (cells or groups of cells) (Moon et al., 2018). Different path-finding algorithms have been implemented and several of them rely on knowing *a priori* the location of the “root cell” (Bendall et al., 2014; Shin et al., 2015; Matsumoto and Kiryu, 2016; Setty et al., 2016; Welch, Hartemink and Prins, 2016). There are also methods that do not require *a priori* knowledge and generate the longest path connecting all nodes in the graph (Trapnell et al., 2014; Ji and Ji, 2016). The resulting graphs can be represented using linear trajectories, showing for example the differentiation trajectory of a progenitor cell towards an intermediate state, and then ending in a differentiated stage (Deconinck et al., 2021). Methods such as SCORPIUS (Cannoodt et al., 2016) and MATCHER (Welch et al., 2017) are specific to linear trajectories. Likewise, cyclical trajectories (for cell cycle modeling, for example) can be inferred from methods like ElPiGraph (Albergante et al., 2020). Nevertheless, cell differentiation in some developmental events is not linear, and may include branching points (Deconinck et al., 2021); therefore, Slingshot (Street et al., 2018) and Monocle (Trapnell et al., 2014) are commonly used methods for modeling this type of events. Meanwhile, recent methods such as PAGA (Wolf et al., 2019) or TinGa (Todorov et al., 2020) are able to extend the modeling trajectory to allow the inclusion of loops or even separated trajectories. Finally, other approaches that model developmental processes in a probabilistic manner to infer trajectories are also available. One of them is Ouija, an approach that uses Bayesian probability to learn pseudo time from a small set of known or suspected marker genes (Campbell and Yau, 2019). SCORPIUS, MATCHER, ElPiGraph, Slingshot, Monocle, PAGA, and Ouija methods are included in the collection of R packages dynverse (https://dynverse.org/), which is also useful for comparing trajectory inference methods to select the most suitable method (Saelens et al., 2019).

### Kinetics of transcription

Additional information can be obtained from cells’ mRNA lifecycle kinetics to infer the state of a cell before and after the time of measurement. Researchers have demonstrated in bulk RNA-seq that transcriptional data can be used to quantify the relative abundance of mRNA molecules at different lifecycle stages (Zeisel *et al.*, 2011). Authors applied this idea to scRNA-seq in an mRNA kinetic model called RNA velocity, where the time derivative of gene expression is calculated (La Manno *et al.*, 2018). RNA velocity distinguishes between nascent pre-mRNAs (unspliced) and mature mRNAs (spliced) from single-cell datasets. Thus, RNA velocity can quantify cellular transitions and reveal transient cellular states in a heterogeneous population. Moreover, it has been demonstrated thar RNA velocity information can be also useful for trajectory inference (Zhang and Zhang, 2021). An overview of the challenges of RNA velocity modeling is discussed in (Bergen *et al.*, 2021).

### Gene regulatory networks

Numerous computational methods have been developed to infer gene regulatory networks (GRN) using single-cell datasets (Pratapa et al., 2020). GRN have been used to identify cell types and states (Moignard et al., 2015; Aibar et al., 2017; Chan, Stumpf and Babtie, 2017; van Dijk et al., 2018). The underlying principle of GRN is that the coordinated function of a limited number of transcription factors and co-factors regulates numerous downstream targets determining the transcriptional state of a cell. Graphs are used for the visual representation of GRNs where nodes are genes and edges are interactions (activation or inhibition). These graphs are undirected since edges are bidirectional. However, given the inherent stochastic variations (e.g. transcriptional bursting), sparsity of gene expression at single-cell resolution, drop-outs, and technical variations, deriving GRN from single-cell datasets is challenging (Raj and van Oudenaarden, 2008; Grün, Kester and van Oudenaarden, 2014; Stegle, Teichmann and Marioni, 2015). One of the main approaches of these methods is inferring networks through gene co-expression. For example, Single-Cell rEgulatory Network Inference and Clustering (SCENIC) finds modules of genes co-expressed with transcription factors and filters them to retain only those modules in which target genes have significant motif enrichment (Aibar et al., 2017). Pratapa et al. recently performed a systematic evaluation of 12 algorithms for inferring GRNs using synthetic networks, datasets from curated models from literature, and datasets from experimental single-cell transcriptomics (Pratapa et al., 2020). The authors published a series of recommendations and implemented a framework (https://github.com/murali-group/BEELINE) to evaluate the selected GRN methods.

## Single-cell/nuclei sequencing for understanding Alzheimer’s disease

Alzheimer’s disease (AD) is the most common cause of dementia worldwide with a prevalence expected to reach 113 million by 2050 (Wu et al., 2017). AD is clinically characterized by a progressive decline in memory, language, problem-solving and cognitive skills (Dubois et al., 2010). The core pathological hallmarks of AD are the extracellular deposition of *β*-amyloid (Aβ) plaques and the accumulation of neuronal fibrillary tangles (NFTs). NFTs are aggregates of Tau protein and Apolipoprotein E. The accumulation of Aβ plaques and NFTs is cytotoxic and causes synapse and neuron loss. Consequently, another pathological hallmark of AD is a widespread neuronal loss specifically in the cortex and hippocampus (McKhann et al., 1984). Increasing evidence suggests that neuroinflammation is also a major contributor to the pathogenesis of AD (Heneka et al., 2015). Pro-inflammatory cytokines, markers of reactive astrocytes and activated microglia, have been found in AD brains of both postmortem humans and transgenic mouse models (Bamberger et al., 2003; Olabarria et al., 2010; Medeiros and LaFerla, 2013). However, the use of anti-inflammatory drugs to treat AD has been unsuccessful mainly because both microglial activation and reactive astrogliosis are complex multi-stage processes that yield diverse phenotypes (Monterey et al., 2021). Thus, it is evident that neuroinflammation is a complex response that involves diverse cell types (neurons, astrocytes, and microglia) with different molecular alterations as well as the crosstalk between them.

Microarray and bulk RNA-seq studies of AD brains have revealed downregulation of neuronal functions and upregulation of immune responses (Heneka et al., 2015; Canter, Penney and Tsai, 2016; De Strooper and Karran, 2016; Nativio et al., 2018). Thus, interactions between the triad (neurons, astrocytes, and microglia) are an important part of the pathophysiology of AD. Other pathways found to be dysregulated are energy metabolism, synaptic transmission, myelin-axon interactions, cytoskeletal dynamics, and protein misfolding (Ginsberg et al., 2000; Colangelo et al., 2002; Blalock et al., 2004; Miller, Oldham and Geschwind, 2008). It is important to note that AD pathology starts in brain regions involved in learning, memory, perception, and emotion (entorhinal cortex, amygdala, and hippocampus) and progresses throughout the cortex (Braak and Braak, 1991, 1996). Interestingly, the hippocampus depicts regional vulnerability with CA1 pyramidal neurons more severely affected than CA2, CA3, and CA4 neighbors (Padurariu et al., 2012). Dissecting the cellular identities and correlating with their vulnerability to degeneration and their contribution to neuroinflammation is fundamental for understanding AD pathogenesis and it can open new avenues for the development of therapeutics. Here, we review recent pioneering single-cell and single-nuclei human transcriptomic studies aimed at studying the cellular heterogeneity involved in Alzheimer’s disease (Table 1).

**TABLE 1:** Selected examples of recent scRNA-seq or snRNA-seq studies in AD. Prefrontal cortex (PFC), Entorhinal cortex (ECtx), Dorsolateral prefrontal cortex (DLPFC), temporal neocortex (TNC), parietal lobe (PL), and superior frontal gyrus (SFG). Oligodendrocyte precursor cells (OPC), oligodendrocytes (Oligo), astrocytes (Astro), microglia (Mgl), inhibitory neurons (InN), excitatory neurons (ExN), and endothelial cells (EC).

Source	Brain region	Cell type addressed	Condition	Number of cells (nuclei) analyzed	Subpopulations found	Single nuclei or cell	Major findings	Ref.
Frozen postmortem brain	PFC	All cells	24 controls and 24 age-matched individuals with varying degrees of AD	80,660	41 clusters: 13 ExN, 12 InN, 4 Astro, 5 Oligo, 3 OPC, 4 Mgl	Nuclei	Gene expression in ExN, InN, Astro, Oligo and Mgl is dysregulated in AD brains	Mathys *et al.* (2019)
Frozen postmortem brain	ECtx	All cells	6 AD individuals and 6 sex and age-matched controls	13,214	8 clusters: MGL, Astro, neurons, Oligo, OPC, EC, hybrid, and unidentified	Nuclei	Astro, EC, and Mgl showed the highest gene expression differences between controls and AD	Grubman *et al.* (2019)
Frozen postmortem brain	ECtx, SFG	All cells	10 individuals with different Braak stages (0, 2, and 6)	106,136	7 broad clusters (ExN, InN, Astro, Oligo, OPC, Mgl, and EC) with 9 and 11 specific subpopulations of ExN in the ECtx and SFG	Nuclei	Some subpopulations of ExN in the ECtx and SFG are more susceptible to degeneration	Leng *et al.* (2021)
Autopsy brains and surgical resection	DLPFC and TNC	Mgl	3 controls, 4 MCI, and 10 AD individuals	16,242	9 Mgl subpopulations	Cell	Mgl subtypes are altered in AD brain	Olah *et al.* (2020)
Frozen postmortem brain	PL	All cells	2 individuals with sporadic AD and 1 carrying a mutation in PSEN1	30,000	14 clusters of cells: 6 ExN, 2 InN, Astro, Oligo, OPC, EC, Mgl	Nuclei	ExN proportion is reduced in samples of sporadic AD	Del-Aguila *et al.* (2019)

There are several limitations to performing scRNA-seq/snRNA-seq from frozen postmortem brains, for example, the difficulty of acquiring postmortem brain samples and the low quality due to the degradation of mRNA. The quality of RNA is typically evaluated using the RNA integrity number (RIN), which is the result of numerous factors, including postmortem interval (PMI: time between death and brain processing), patient’s medical condition previous to death, storage conditions, as well as storage time (White et al., 2018). The mRNA degradation is tissue-specific, transcript-specific, and molecule-specific (Nagy et al., 2015; Sobue et al., 2016; Zhu et al., 2017). As expected, transcripts with a lower expression will degrade faster; this is why performing snRNA-seq on postmortem brains is more challenging than bulk RNA-seq. Studies have also demonstrated that miRNAs have a higher PMI-dependent resistance to degradation than other biomolecules (Nagy et al., 2015). There is no clear correlation between the PMI and RIN values obtained from postmortem brain samples. Thus, the general recommendation when requesting tissue from a brain bank is to require similar RIN values, rather than PMI.

Severely affected brain regions in AD are characterized by massive neuronal loss. Therefore, sequencing regions with a high neuronal deficit may bias cell proportions since they rely on assigning cells to clusters that might not have a correct representation. This problem was solved by Mathys et al. in one of the first studies that provided insights into the heterogeneity of AD in cortical regions with single-cell resolution (Mathys et al., 2019). Researchers profiled 80,660 nuclei isolated from prefrontal cortices obtained from 48 human postmortem brains with varying degrees of AD pathology, including controls. Interestingly, the analysis of cell profiles from all combined samples yielded cell types, markers, and cell-type proportions highly consistent with another human cortex snRNA-seq study of neurotypical controls (Lake et al., 2018). Mathys et al. found a total of 8 major transcriptional clusters(each with subclusters) including excitatory neurons, inhibitory neurons, astrocytes, oligodendrocytes, microglia, oligodendrocyte precursor cells (OPC), endothelial cells, and pericytes. When comparing samples with AD-pathology against no-pathology, 1031 DEGs were found in total in different cell types (Mathys et al., 2019). DEGs specific to excitatory and inhibitory neurons were mostly downregulated whereas oligodendrocytes, astrocytes, and microglia depicted more than 50% of upregulated DEGs. Notably, authors found transcriptional dysregulation due to AD pathology in all cell types, with sex-dependent differences. Dysregulation was highest in the early AD stages, and it was mostly cell-type-specific. Conversely, in late AD stages, upregulated DEGs were common to several cell types denoting a global response (Mathys et al., 2019).

One of the first cortical regions that depict neurofibrillary inclusions and neuronal loss at the early stages of AD is the entorhinal cortex. Grubman et al. characterized the heterogeneity underlying this early affected region using postmortem human brains (Grubman et al., 2019). Authors sequenced 13,214 nuclei isolated from entorhinal cortex tissue, dissected from 6 AD individuals and 6 sex and age-matched controls. Their results demonstrated the presence of all 6 known brain cell types (microglia, astrocytes, neurons, OPC, oligodendrocytes, and endothelial cells) of which astrocytes, endothelial cells, and microglia showed the highest gene expression differences between controls and AD. Researchers also demonstrated that specific transcription factors have alterations in opposite directions in different cell subpopulations. For example, in AD, APOE was found downregulated in OPC and upregulated in astrocytes and microglia (Grubman et al., 2019). This is an example of the advantage of using snRNA-seq over bulk RNA-seq; it is possible to detect transcripts with opposite regulation.

Damage to the parietal lobe is common in the early stages of AD, however, its role in the development of AD remains elusive. Del-Aguila et al. transcriptionally profiled single-nuclei isolated from the parietal lobes of an individual carrying a known mutation in PSEN1 gene (p.A79V) and 2 relatives with sporadic AD (Del-Aguila et al., 2019). Samples were obtained from postmortem brains and a total of 30,000 nuclei were sequenced. Authors identified and annotated 14 clusters including 6 subclasses of excitatory neurons, 2 inhibitory neurons, oligodendrocytes, astrocytes, microglia, OPC, and endothelial cells. A reduced proportion of excitatory neurons was observed after comparing the proportion of cells in each cluster between the mutation carrier sample against the sporadic AD samples (Del-Aguila et al., 2019). Furthermore, Del-Aguila et al. performed a pseudo time gene expression reconstruction using TSCAN (Ji and Ji, 2016). This method was implemented using the transcriptomic profile of microglial cells and it allowed authors to capture the sequence of activation and transition to disease-associated microglia (DAM) cells. The expression profiles of DAM cells were obtained from a previous study (Keren-Shaul et al., 2017). Interestingly, the authors found that 79 genes of the DAM markers were significantly associated with a temporal trajectory. This is the first study that analyzed DAM markers in single-nuclei microglia profiles obtained from AD brains carrying a known mutation.

A recent pioneering study adopted an interesting strategy to delineate the progression of AD in specific cell types (Leng et al., 2021). Leng et al. isolated 42,528 and 63,608 single nuclei from the entorhinal cortex and superior frontal gyrus respectively; samples were obtained from 10 human postmortem brains with varying degrees of AD-tau neuropathological progression (inferred from the Braak stage). The entorhinal cortex and superior frontal gyrus regions were selected because they are affected in the early and late stages of AD correspondingly (Braak and Braak, 1991). Leng et al. found 9 and 11 subpopulations of excitatory neurons in the entorhinal cortex and the superior frontal gyrus respectively depicting region-specific genes. Authors found subpopulations of neurons in both regions which were more vulnerable to degeneration in the early and late stages. Furthermore, marker genes potentially responsible for these selective vulnerabilities were derived and validated. For example, the relative abundance of a subpopulation of excitatory neurons in the entorhinal cortex showed a striking decrease in Braak stage 2 samples compared to Braak stage 0, suggesting their susceptibility to neurodegeneration in early stages (Leng et al., 2021). By correlating the degree of degeneration with the region, cell subpopulations, transcriptional profiles, and relative abundances, researchers were able to determine subpopulation selective vulnerabilities.

Given that mounting evidence suggests a microglial role in aging and AD pathology, Olah et al. profiled 13,368 single live cells isolated from the dorsolateral prefrontal cortices of autopsy samples from individuals with mild cognitive impairment or AD pathology (Olah et al., 2020). Additionally, 2,874 cells used as controls were isolated from temporal neocortices of individuals undergoing intractable epilepsy surgeries. The authors used a previously published protocol involving FACS to purify microglial cells using antibodies for CD11b and CD45 (Olah et al., 2018). Single cells were isolated using droplet technology (Olah et al., 2020). Interestingly, researchers found 9 distinct microglial clusters and validated 4 of them histologically using marker genes. Since samples were obtained from individuals without dementia, the authors used a gene set enrichment approach to link microglial subclusters to diseases. One microglial cluster was found to be altered in AD and it was validated in the single nucleus RNA-seq study by Mathys et al. (Mathys et al., 2019).

## Single-cell/nuclei sequencing for understanding Parkinson’s disease

Parkinson’s disease (PD) is one of the most common slowly progressive neurodegenerative movement disorders but is very challenging at advanced stages. A histological hallmark of PD is the accumulation of fibrillar aggregates called Lewy bodies, enriched in α - Synuclein misfolded protein (Wakabayashi et al., 2007). Another important characteristic of PD is the degeneration of dopaminergic neurons (DaNs) in the *substantia nigra pars compacta* (SNpc). Loss of these neurons leads to PD motor symptoms such as rigidity, resting tremor, slowness in movement (bradykinesia), and postural instability (Bloem, Okun and Klein, 2021).

Midbrain DaNs have important roles in the regulation of voluntary movement, reward, and emotion. These brain cells are highly heterogeneous even though they share a common neurotransmitter phenotype and lie in close proximity within the ventral midbrain. The current classification of DaNs is based on topographical features, for example, anatomical location and axonal innervation targets. Traditionally, three distinct types of midbrain DaNs are considered: A8, A9, and A10 located in the retrorubral field (RRF), SNpc, and ventral tegmental (VTA) areas respectively (Bentivoglio and Morelli, 2005). DaNs depict heterogeneous susceptibilities to neurodegeneration, specifically to PD. A9 DaNs project their axons into the dorsal striatum through the nigrostriatal pathway, and they are involved in the control of involuntary movement. Although A9 DaNs are the primarily degenerated cell type in PD (Lees, Hardy and Revesz, 2009), other cell types such as astrocytes and microglia have also been implicated in neurodegeneration and PD pathogenesis [reviewed in (Brück et al., 2016; Booth, Hirst and Wade-Martins, 2017)]. A8 and A10 DaNs depict projections into the ventral striatum and the prefrontal cortex, and they are involved in the regulation of emotion and reward. Degeneration of A8 and A10 DaNs is associated with schizophrenia, drug addiction, and depression (Tzschentke and Schmidt, 2000), highlighting the different functions of dopamine in specific brain regions. An important characteristic of human DaNs is that they have up to one million axon terminals, many of which reach long distances from the soma, with projections to the putamen, and the caudate nuclei, compared to neocortical neurons which have up to tens of thousands of synapses (Bolam and Pissadaki, 2012). Thus, this uniquely massive, unmyelinated axonal branching renders ventral midbrain DaNs under a high energy demand compared to neurons from other regions contributing to their vulnerability (Pissadaki and Bolam, 2013). The molecular mechanisms that underlie the phenotypic and functional differences between ventral midbrain DaNs are largely unknown. Limited access to all these brain regions makes single-cell studies experimentally challenging. Here, we review recent pioneering single-cell and single-nuclei human transcriptomic studies aimed at elucidating the cellular heterogeneity involved in Parkinson’s disease (Table 2).

**TABLE 2:** Selected examples of recent scRNA-seq or snRNA-seq studies in PD. Middle frontal gyrus (MFG), and substantia nigra (SN). Oligodendrocyte precursor cells (OPC), oligodendrocytes (Oligo), astrocytes (Astro), microglia (Mgl), macrophages (Macro), inhibitory neurons (InN), excitatory neurons (ExN), endothelial cells (EC), dopaminergic neurons (DaNs), fibroblasts (Fibro), mural cells (MC), pericytes (Peri), neuroblasts (NB) and young neurons (yNeu).

Source	Brain region	Cell type addressed	Condition	Number of cells (nuclei) analyzed	Number of subpopulations found	Single cell or nuclei	Major findings	Ref.
Frozen postmor-tem brains	MFG and SN	All cells	5 controls	16,649	10 clusters in SN: 2 Astro, 3 Oligo, EC, Mgl, OPC, DaNs, and GABAergic neurons	Nuclei	Gene expression of DaNs and Oligo from the SN are associated to PD genetic risks	Agarwal *et al. (2020)*
Frozen postmor-tem brains	SN	All cells	7 controls	44,274	24 clusters: 3 Astro, Fibro, MC, EC, 3 Mgl, 7 neurons including DaNs, 5 Oligo, 2 OPC	Nuclei	Cell-type-specific expression patterns in mouse SN are highly similar to human SN	Welch *et al.* (2019)
Frozen postmor-tem brains	Mid-brain	All cells	6 idiopathic PD individuals and 5 age and sex-matched controls	41,435	12 clusters: 5 neurons (InN, ExN, GABAergic, DaNs, CADPS2 high), OPCs, Oligo, Astro, ependymal, Peri, EC, and Mgl	Nuclei	Dysregulation of gene expression is observed in neuronal and glial cells of brains with idiopathic PD	Smajić *et al.* (2021)
Frozen postmor-tem brains	Mid-brain	All cells	8 controls and 10 age-matched and PMI-matched individuals with PD or Lewy body dementia	387,483	10 clusters of DaNs in controls, and 68 in PD including DaNs, ExN, InN, Astro, OPC, Oligo, EC/Peri, and Mgl/Macro	Nuclei	Some DaNs subtypes are more susceptible to degeneration than others	Kamath *et al.* (2022)
iPSC	None	DaNs	WT and SNCA-A53T mutant iPSC differentiated into DaNs	15,325	6 clusters: 2 neuron progenitors and 4 DaNs	Cells	DaNs have different degrees of sensitivity	Fernandes *et al.* (2020)
iPSC	None	TH + DaNs	3 WT and 2 PD individuals with GBA-N370S mutation	146	Not applicable	Cells	HDAC4 is the common early repressor of downregulated genes in PD	Lang *et al.* (2019)
iPSC midbrain organoids	None	All cells	1 WT and a cell line with isogenic LRRK2-p.Gly2019Ser insertion	10,475	8 clusters: NB, yNeu, DaNs, non-DaNs, glia, progenitors, Peri, EC	Cells	PD-related mutations disrupt midbrain development	Zagare *et al.* (2022)

Researchers have contributed to profiling the cellular heterogeneity with single-cell resolution of the SN using postmortem brains of both healthy and PD individuals. One of the earliest single-nuclei transcriptomic studies of the SN was published by Agarwal et al. (Agarwal et al., 2020) who sequenced 10,706 and 5,943 nuclei from the middle frontal gyrus and SN, respectively. Researchers obtained 12 region-matched samples from 5 human postmortem brains without neurological disease. A transcriptomic cellular atlas was compiled from the identification of cell-type-specific gene expression patterns in 10 distinct cell subpopulations found in SN: 2 types of astrocytes, 3 subtypes of oligodendrocytes, endothelial cells, microglia cells, oligodendrocyte precursor cells, DaNs, and GABAergic neurons. Significant associations were found between PD genetic risks (obtained from GWAS) and specific SN subpopulation gene profiles (DaNs and oligodendrocytes) (Agarwal et al., 2020).

In another study, researchers developed a computational method called LIGER (linked inference of genomic experimental relationships) to enable the combination and analysis of single-cell datasets from different individuals, species, regions, conditions, and molecular origin (genomic, epigenomic, and spatial) (Welch et al., 2019). To test their method, Welch et al. generated snRNA-seq from 44,274 single-nuclei isolated from the SN of 7 frozen postmortem brains (healthy controls). A total of 24 cell clusters were identified: 3 subtypes of astrocytes, fibroblasts, mural cells, endothelial cells, 3 microglia types, 7 neuron classes including DaNs, 5 oligodendrocytes, and 2 oligodendrocyte precursor cell subtypes (Welch et al., 2019). Next, the authors compared their SN dataset with a published single-cell dataset obtained from the SN of healthy mice (Saunders et al., 2018) and found a high correlation in the cell-type-specific expression patterns between these species. Gene ontology (GO) analysis of highly correlated human-mouse gene pairs yielded significantly enriched gene sets related to brain cell identity and molecular functions (Welch et al., 2019). Conversely, gene pairs with the lowest correlation were enriched in DNA repair and chromatin remodeling functions, suggesting species differences in epigenetic regulatory functions (Welch et al., 2019). Although this comparison between species was performed on healthy individuals, the approach proposed by this study can be implemented in future studies to compare samples obtained from rodent models of PD with samples obtained from PD human patients (Duty and Jenner, 2011). Moreover, regarding the comparison between species, Geirsdottir et al. demonstrated through single-cell analysis and by using ortholog conjectures that human microglial cells depict significant heterogeneity when compared among other vertebrate species, and in addition, microglia-specific gene expression profiles depicted significant susceptibility for PD and AD (derived from human GWAS) in primates and humans (Geirsdottir et al., 2019).

Additionally, in a recent study, researchers profiled 41,435 single nuclei from postmortem midbrains of 6 individuals diagnosed with idiopathic PD (IPD) and 5 age and sex-matched controls (Smajić et al., 2021). In this study Smajic et al. demonstrated that the dysregulation in IPD is not exclusive to DaNs of the SN, but also different cell types show alterations in other brain regions. Authors found a neuronal cell cluster present only in IPD midbrains, concomitant to reduced numbers of oligodendrocytes in IPD samples. Moreover, an increased microglial subpopulation of the SN in IPD depicted an amoeboid shape, suggesting an activated state (Smajić et al., 2021).

Another comprehensive study recently available was performed by Kamath et al., 2021, (Kamath et al., 2022). This is the first study that profiled hundreds of thousands of nuclei (184,673 and 202,810) obtained from postmortem midbrains of both controls and individuals diagnosed with PD or Lewy body dementia (LBD). Researchers purified nuclei using fluorescence-activated nuclei sorting (FANS) and a nuclear receptor (NURR1 or NR4A2) previously found to be essential for the survival of DaNs in mice (Zetterström et al., 1997; Saunders et al., 2018). The deep sequencing strategy and the data analysis method (LIGER) used allowed researchers to identify 10 clusters of DaNs in both controls and PD/LBD individuals. Interestingly, the authors used a method to accurately determine cell proportions thus, eliminating technical confounders, for example, batch effects and individual variations (MASC: mixed effect association of single cells), and found that one of the DaNs clusters was significantly reduced in PD samples, while another one was increased. These observations were validated through single-molecule fluorescence *in situ* hybridization (smFISH). Overall, these results confirm the existence of diverse subpopulations of DaNs with different degeneration susceptibilities.

The development of iPSCs and their application for disease modeling has resulted in powerful methodologies to study human complex pathologies. Reprogramming somatic cells from individuals carrying known PD-related mutations to iPSCs has been used to differentiate DaNs *in vitro*, generating a valuable source of cells that would otherwise be accessible only from postmortem brains, where the progression of PD is already at its endpoint. Thus, modeling PD *in vitro* through the differentiation of iPSCs to form DaNs, allows the discovery of biomarkers at the onset of disease, and facilitates drug testing (Laperle et al., 2020). However, one limitation of such studies is the high degree of heterogeneity and cellular variability. Variability has been reported in several aspects including iPSC differentiation potential, DNA methylation, transcriptional profiles, and even cell morphologies due to differences between donors, experiments, and genetic stability (Kilpinen et al., 2017; Volpato and Webber, 2020). To overcome these limitations, scRNA-seq and snRNA-seq are currently being used first, to compare the similarity of cells differentiated from iPSCs with those found *in vivo*, and then to elucidate the cellular heterogeneity at the onset of PD by using dopaminergic neurons differentiated from iPSCs. In this context, La Manno et al. demonstrated through scRNA-seq that dopaminergic neurons generated from human iPSC recapitulated key stages of *in vivo* midbrain development, retained the expression of markers genes, and conserved the cellular heterogeneity observed *in vivo* (La Manno et al., 2016).

In a recent study by Fernandes et al., researchers generated a common PD mutation (A53T in SNCA gene) in a human iPSC line (Human induced pluripotent stem cell initiative, Sanger Institute) and induced both WT and A53T-SNCA iPSC into DaNs using a widely accepted induction protocol (Fernandes et al., 2020). After 6 weeks, more than 15,000 cells were profiled from scRNA-seq, and using the expression of known marker genes, 6 clusters were found: 2 neuronal progenitors, and 4 dopaminergic neuron subpopulations. Also, authors found overlapping clusters between their clusters and those found in a study of substantia nigra from 7 human adult postmortem brains (Welch et al., 2019), particularly in clusters characterized by the positive expression of Tyrosine Hydroxylase, thus suggesting that *in vitro* DaNs are representative of corresponding *in vivo* DaNs. Finally, induced cells were tested with cytotoxic drugs and genetic stressors and the proportion of cells in clusters changed, suggesting that clusters presented varying degrees of sensitivity (Fernandes et al., 2020).

To overcome the difficulties inherent to cellular heterogeneity of iPSC neuronal induction, Lang et al. sorted iPSC-derived DaNs using FACS with the Tyrosine Hydroxylase intracellular marker (Lang et al., 2019). The authors derived iPSCs and performed both bulk and single-cell RNA-seq from two individuals carrying a known PD mutation (GBA-N370S) and three controls. Although the number of profiled cells after sorting was reduced (146 cells), authors were able to identify DEGs. Notably, cells segregated between control and PD along the second component of the PCA plot. By combining DEGs found with bulk and scRNA-seq and clustering single cells with the Single-cell consensus clustering (SC3) method (Kiselev et al., 2017) authors obtained a core set of 60 DEGs, with the majority downregulated in PD. Using this small set of core genes, the authors implemented the Ouija method to infer single-cell pseudo times (Campbell and Yau, 2019). As a result, authors suggested a “continuous disease axis” of gene expression variation with 60 core genes mostly downregulated. The authors used ingenuity pathway analysis (IPA) (Krämer et al., 2014) to build regulatory gene networks and found that the Histone deacetylase HDAC4 was the common early repressor of the core set of genes. Pharmacological modulation of HDAC4 rescued PD-related phenotypes, including ER stress (Lang et al., 2019). These results show that even with a low number of cells, it is possible to find molecular drivers of a specific PD phenotype, through purification before sequencing, and a comprehensive data analysis using the appropriate computational methods.

Cerebral organoids have emerged as a useful 3D model for neural differentiation studies since they recapitulate certain aspects of brain development and disease-associated phenotypes (Lancaster et al., 2013). For example, by comparing neural precursors differentiated in 2D *vs*. 3D by scRNA-seq, it was recently established that midbrain organoids presented lower levels of cellular senescence and mitochondrial stress, which correlated with resistance to toxic challenges, robust synaptic contacts, and functionality of DaNs (Kim et al., 2021). Furthermore, another study found by single-cell analysis demonstrated that midbrain organoids maintain the same neuronal heterogeneity observed *in vivo*, indicating that organoids are a useful system for the *in vitro* study of neurodegenerative diseases (Smits et al., 2020). Thus, pathological features have been analyzed in brain organoids generated from isogenic iPSCs that carry a PD-related mutation in LRRK2. In such context, scRNA-seq experiments showed clear differences between the normal and mutated iPSCs. The LRRK2 p.Gly2019Ser mutation disrupted normal development, resulting in incomplete differentiation and reduced viability (Zagare et al., 2022). These studies show that midbrain organoids and scRNA-seq constitute an excellent system to study different cellular and molecular aspects related to PD.

## Discussion

In the last decade, impressive progress has been achieved through the use of scRNA-seq/snRNA-seq to elucidate the transcriptional and regional heterogeneity of brain cells. These developments have transformed our understanding of brain cell diversity and their interplay in neurodegenerative disease conditions. Even though methods for scRNA-seq/snRNA-seq have been well developed, several limitations and challenges remain. One example is the source of brain cells. Fresh brain samples mainly from resection surgeries are scarce and the best alternatives are either postmortem brains or differentiated iPSCs. The integrity of the nuclear transcripts obtained from postmortem brains must be carefully determined and brain samples should have similar RIN values. Also, it is important to have access to brain donors’ clinical information including the degree of neurodegeneration to adequately form groups or model covariables. A well-designed experiment including age, sex, and degree of degeneration-matched controls is very important to reduce the number of variables affecting gene expression.

Additionally, since AD and PD are characterized by progressive neurodegeneration and loss of neurons in different regions, identifying the cell subtypes whose proportions are altered in AD or PD is challenging. Researchers rely on algorithms that automatically preprocess and normalize the datasets assigning cells to clusters. However, different algorithms or parameter choices can lead to divergent results. Careful consideration must be employed when selecting which methods to use for data analysis and experimental validation may be needed. Furthermore, the preparation of single cells or nuclei in suspension is also a major challenge when sequencing transcripts obtained from brain cells or nuclei. Particularly for single-cell suspensions, it is difficult to isolate intact brain cells because they are embedded in a complex and interconnected network. Also, neurons are very sensitive to cell dissociation methods, therefore scRNA-seq datasets may show an underrepresentation of neuronal types with respect to glial cells (Darmanis et al., 2015). Therefore, since nuclei isolation protocols are faster and they represent the best alternative when the tissue is degraded, frozen or formalin-fixed (Lacar et al., 2016), snRNA-seq is an excellent alternative for profiling of brain cells. In addition, studies have demonstrated that snRNA-seq results are comparable to scRNA-seq results (Bakken et al., 2018).

Another important caveat of scRNA-seq and snRNA-seq is that by isolating cells or nuclei, their spatial information and connectivity are lost. Transcriptional profiles can convey information about a cell’s identity and the interplay with other cells, however, the information about neighboring cells or their proximity to pathologies (e.g. tau fibrillary tangles, Lewy bodies) is unknown. To address this deficiency, an emerging high-throughput alternative is spatial transcriptomics (Ståhl et al., 2016). Spatial transcriptomic methods are now being used to resolve the location of cells, for example, smFISH and Slide-Seq (Raj et al., 2008; Rodriques et al., 2019). However, it is important to consider that through spatial transcriptomics it is difficult to obtain conclusions with single-cell resolution because these methods consist of profiling spots distributed throughout a histological sample and each spot covers various cells (Noel et al., 2022). Furthermore, the analysis of spatial transcriptomics data can be challenging since detailed computational methods for their study do not exist or are still in development (Atta and Fan, 2021). Thus, in the near future, scRNA-seq/snRNA-seq technologies should be combined with other multi-omics and with spatial transcriptomics to provide a holistic understanding.

Overall, AD and PD brain scRNA-seq and snRNA-seq are providing new insights, for example, identifying cell subpopulations that are more vulnerable to degeneration and cellular transcriptional profiles specific to the affected regions. Particularly, the identification of cell subpopulations and the overall characterization of cell heterogeneity in brain regions affected by AD or PD is valuable for understanding why some neuronal or glial cell types are more susceptible to degeneration than others, as well to better understand how gene expression is regulated at the single-cell level in a pathological state of the brain. Besides, the comparison of the information obtained from single-cell datasets with data obtained, for example, from GWAS, can provide insights for the identification of specific cell subtypes or genes that may provide the risk of neurodegenerative diseases development. The construction of cell trajectories can show the progression of cellular degeneration as the disease progresses, and infer cell types that are lost while others are enriched. Thus, future advancements in this area will facilitate the discovery of potential and novel therapeutic targets.
